# Human urothelial carcinoma cell response to Sunitinib malate therapy *in vitro*

**DOI:** 10.1186/s12935-015-0179-z

**Published:** 2015-02-28

**Authors:** Jin Wen, Han-Zhong Li, Zhi-Gang Ji, Jing Jin

**Affiliations:** Department of Urology, Peking Union Medical College Hospital, Chinese Academy of Medical Sciences & Peking Union Medical College, Shuai Fu Yuan 1, Wang Fu Jin Street, Beijing, 100730 China; Institute of Materia Medica, Chinese Academy of Medical Sciences & Peking Union Medical College, Beijing, China

**Keywords:** Sunitinib, Sorafenib, Cisplatin, Bladder cancer, Proliferation

## Abstract

**Objectives:**

Bladder transitional cell carcinoma (TCC) is one of the most common solid malignancies in China. This study examined the antitumor effect and underlying mechanism of action of sunitinib malate in human bladder TCC *in vitro*.

**Methods:**

Bladder TCC cell lines 5637 and BIU87 were maintained in 1640 medium and T24 cell lines in DMEM/F12 medium. All 3 cell lines were then exposed to graded concentrations (0.625-20 μmol/L) of sunitinib malate, sorafenib and cisplatin for 24–96 hours to determine the sensitivities to each drug. Cell viability was measured by the MTT [3-(4,5-dimethylthiazol-2-yl)-2,5-diphenyltetrazolium] assay, and apoptosis was analyzed by flow cytometry. Cell apoptotic morphology was observed by a fluorescence microscope after DAPI (4′,6-diamidino-2-phenylindole) staining. Protein concentrations were measured by western blot.

**Results:**

Sunitinib malate showed a concentration-dependent inhibitory effect on the 5637, T24 and BIU87 cell lines with IC_50_’s of 1.74 μmol/L, 4.22 μmol/L, and 3.65 μmol/L, respectively. Cisplatin also exhibited good antitumor activity, but whereas sorafenib suppressed proliferation of the cells at concentrations of 10 μmol/L or higher, there was practically no response at lower concentrations. Sunitinib malate treatment resulted in an accumulation of cells in the sub-G1 phase, especially with the T24 and BIU87 cell lines, which induced apoptosis of the cells.

**Conclusions:**

Sunitinib malate exerted marked inhibitory activity against bladder cancer cells. The cell growth inhibitory effect of the drug was related to induction of apoptosis. These results suggest that clinical application of sunitinib-based therapy for advanced bladder cancer is possible.

## Introduction

Bladder transitional cell carcinoma (TCC) is one of the most common solid malignancies in China. Currently, radical cystectomy remains the standard therapy for invasive TCC. However, the disease recurs in up to 50% of patients and is potentially lethal despite surgery. Although bladder TCC is relatively sensitive to GC (gemcitabine, cisplatin) and MVAC (methotrexate, vinblastine, doxorubicin, cisplatin), the response rate with these regimens is no more than 50% and subsequent progression is relatively common. Due to the poor prognosis of advanced bladder carcinoma and the insufficient efficacy of the above therapies, more effective treatments for metastatic and advanced bladder TCC need to be found. Thus, the investigation of novel genetic and pharmacologic agents, including anti-angiogenic agents that can target pathway-specific molecules, has become increasingly important.

As several receptor tyrosine kinases (RTKs), such as the vascular endothelial growth factor (VEGF) receptors, appear to be involved in the development of bladder TCC, RTKs may be attractive targets for therapeutic manipulation. Sunitinib malate is a multi-RTK inhibitor that acts on vascular endothelial growth factor (VEGF) receptors 1, 2, and 3, platelet-derived growth factor (PDGF) receptor, stem cell factor receptor (KIT), and FMS-like tyrosine kinase-3 receptor (FLT3). Its antitumor activity has been demonstrated in other cancers, such as renal cell carcinoma (RCC), gastrointestinal stromal tumor (GIST), non-small-cell lung cancer, and colorectal cancer [1-5]. The objective of this study was to examine the antitumor effect and underlying mechanism of action of sunitinib malate in human bladder TCC *in vitro*.

## Methods

### Cell culture

Human bladder cancer cell lines 5637, T24 and BIU87 were obtained from the Cell Culture Center of the Institute of Basic Medical Science, Chinese Academy of Medical Sciences, and the Cell Culture Center of the Cancer Institute and Hospital, Chinese Academy of Medical Sciences. The 5637 and BIU87 cell lines were maintained in 1640 medium (Gibco, Grand Island, NY, USA) with 10% heat-inactivated newborn calf serum at 37°C in 5% CO_2_, and the T24 cell lines were maintained in DMEM/F12 medium (Gibco, Grand Island, NY, USA) with 10% heat-inactivated newborn calf serum at 37°C in 5% CO_2_.

### Cell viability measurement by MTT assay

Cell viability was measured by the 3-(4,5-dimethylthiazol-2-yl)-2,5-diphenyltetrazolium bromide (MTT) assay. In brief, cells were seeded into 96-well plates at a density of 2 × 10^3^/well. 24 hours later, triplicate wells were treated with media and the test agents (at concentrations of 0.625-20 μmol/L). After incubation for 96 hours at 37°C in 5% CO_2_, the drug-containing medium was removed and replaced by 100 μL fresh medium with 0.5 mg/mL MTT solution. After incubation for 4 hours, the medium with MTT was removed and 150 μL DMSO was added to each well. The plates were then gently agitated until the color reaction was uniform, and the OD570 (optical density at a wavelength of 570 nm) was determined using a microplate reader (Wellscan MK3, Labsystems Dragon). Microsoft® Excel 2003 was used for data analysis. Media-only treated cells served as the indicator of 100% cell viability. The 50% inhibitory concentration (IC_50_) was defined as the concentration that reduced the absorbance of the untreated wells by 50% of that of the vehicle in the MTT assay.

### Apoptosis analysis by flow cytometry (FCM) by PI staining assay and Annexin V-FITC/PI staining assay

For PI staining assay, 5637, T24 and BIU87 cells treated with or without the test agents at various concentrations for 48 hours were collected by trypsinization and washed twice with phosphate-buffered saline (PBS), then fixed in ice-cold 70% (v/v) ethanol at −20°C for 24 hours. After centrifugation, the cell pellets were resuspended in 1 mL of propidium iodide (PI) solution (50 mg/mL PI, 50 mg/mL RNase A, 0.03% Triton X-100, and 0.01% sodium citrate in PBS) and incubated for 30 minutes at 37°C. DNA histograms were obtained by fluorescence-activated cell sorting analysis (FACS). In addition, Annexin V-FITC/PI staining assay was introduced for quantitative determination of apoptotic percentage in T24 cells. After 48 hours treatment, T24 cells were washed with PBS, stained with fluorescein isothiocyanate conjugated Annexin V and PI, according to the manufacturer’s protocol (The ApoTarget™ Annexin-V FITC Apoptosis Kit, Invitrogen, U.S.A.), and analyzed by flow cytometry.

### DAPI (4′,6-diamidino-2-phenylindole) staining

After incubation with or without sunitinib malate for 24 hours, T24 cells were stained with DAPI (10 μg/mL in PBS) for 30 minutes, followed by fixation with 4% PFA (paraformaldehyde) for 15 minutes in the dark. Cell apoptotic morphology was observed by a fluorescence microscope (Olympus BX51, Japan) after washing with PBS.

### Western blot analysis

Total cellular protein was extracted using a lysis buffer [1% Triton X-100 (pH 7.2), 100 μg/ml phenylmethylsulfonyl fluoride, 10 μg/ml leupeptin, 1 μg/mL pepstatin A, 2 μg/mL aprotinin, 20 mM p-nitrophenyl phosphate, 0.5 mM sodium orthovanadate, and 1 mM 4-(2-aminoethyl) benzenesulfonylfluoride hydrochloride]. The protein concentration was measured by the Bio-Rad protein assay. An equal amount of protein was separated using 10% and 12% SDS-PAGE and transferred to nitrocellulose membranes (Amersham, Bucks, UK). The membranes were blocked with 5% skimmed milk in PBS and incubated overnight with primary antibodies, followed by horseradish-peroxidase-conjugated antibodies at room temperature. β-Actin was used as an internal positive control. The primary antibodies included Fas, FasL, PARP [poly (ADP-ribose) polymerase], and β-actin (Santa Cruz Biotechnology, Santa Cruz, CA, USA). Signals were visualized using an enhanced chemiluminescence system (Amersham).

### Wound healing assay

T24 cells were seeded at a density of 10^5^ in 24 well plates and cultured for 24 hours. Monolayers were wounded using the tip of a pipette, washed by PBS, and further incubated in DMEM/F12 medium with 1% FBS in the presence or absence of sunitinib malate at different concentrations for 24 hours. Images were acquired via a phase-contrast microscope and the wound width was measured at various time points.

## Results

### Antitumor effect of sunitinib malate versus that of sorafenib and cisplatin

**Figure 1:**
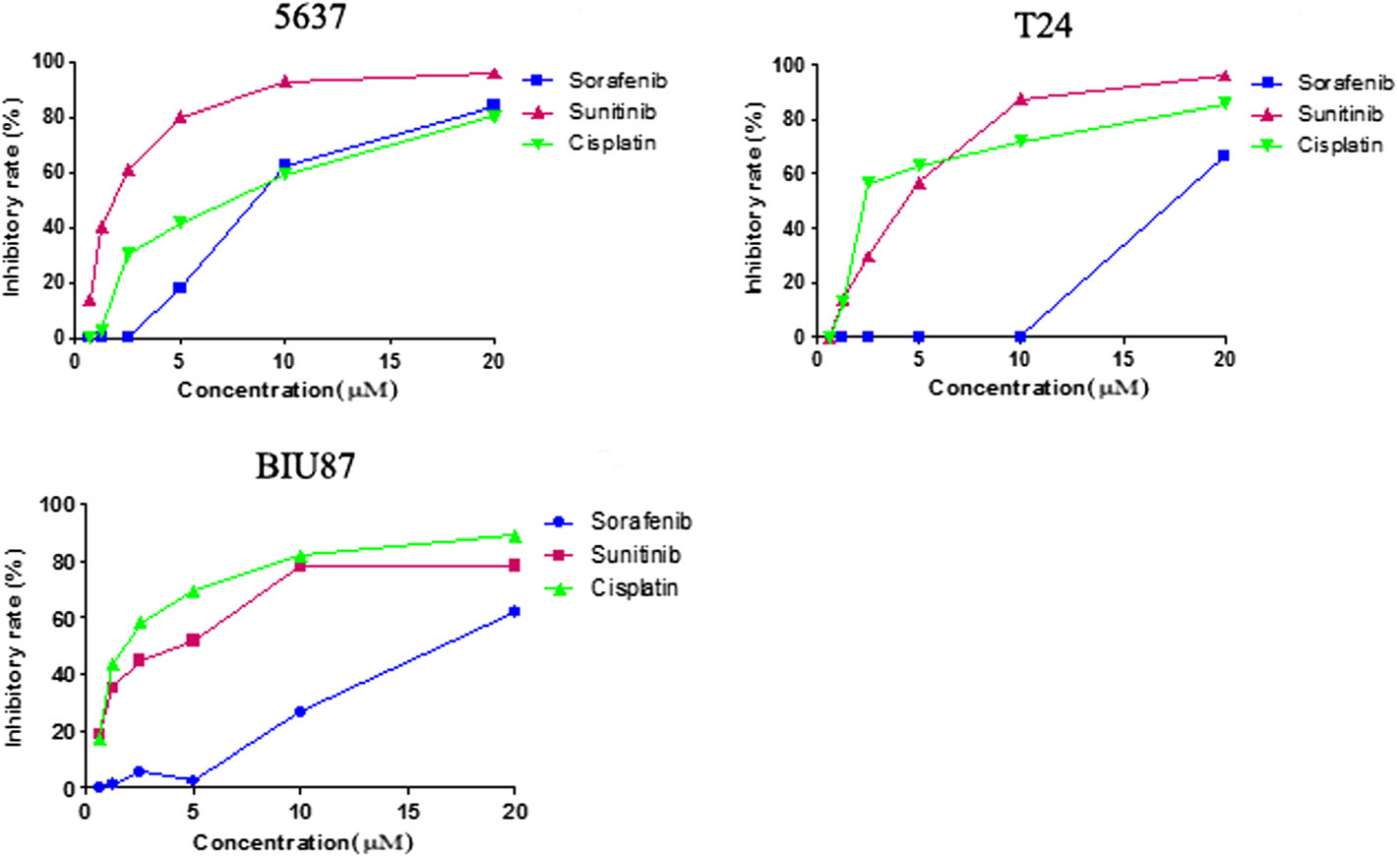
**Antitumor effects of sunitinib malate, sorafenib and cisplatin on the 3 human bladder cancer cell lines following exposure to graded concentrations of 0.625-20 μmol/L of the 3 drugs for 24–96 hours (MTT assay).**

To explore the antiproliferative activity of sunitinib malate on bladder cancer cells, small panels of the 5637, T24 and BIU87 cell lines were initially used in the MTT assay. Since cisplatin is widely used in clinical therapy of bladder cancer, we used it as positive control medicine. In addition, sorafenib, an already launched RTKs inhibitor, was selected as control medicine with similar mechanism of action. As shown in Figure 1, sunitinib malate exerted a concentration-dependent inhibitory effect on the 3 cell lines with 96 h IC_50_’s of 1.74 μmol/L, 4.22 μmol/L, and 3.65 μmol/L, respectively. All 3 cell lines were also exposed to graded concentrations (0.625-20 μmol/L) of sorafenib or cisplatin for 96 hours to determine sensitivities to these drugs (Table 1). At a concentration 10 μmol/L or higher, sorafenib suppressed proliferation of the cancer cells, but at lower concentrations there was practically no response. However, cisplatin also exhibited good antitumor activity (Figure 1).

**Table 1:** **Antiproliferative effect of drugs against bladder cancer cell lines**

**Drug**	**IC**_**50**_ **values (96 h) [μmol/L]**		
	**5637**	**T24**	**BIU87**
Sunitinib	1.74	4.22	3.65
Sorafenib	9.09	15.44	15.08
Cisplatin	6.69	2.87	0.22

### Induction of apoptosis by sunitinib malate

**Figure 2:**
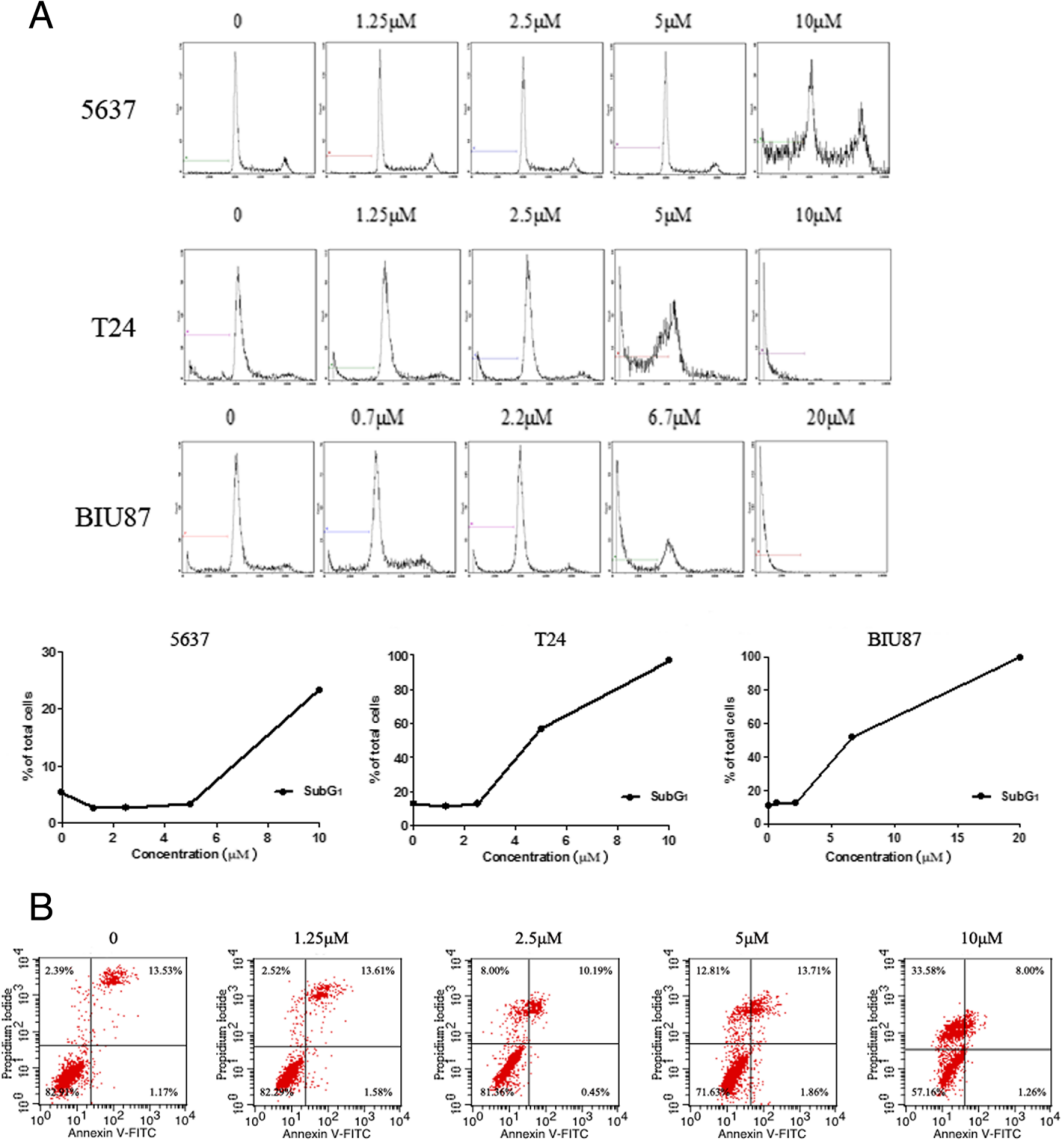
**Apoptosis analysis by flow cytometry. A**, flow cytometric analysis of propidium iodide stained DNA of three bladder cancer cell lines following Sunitinib treatment. Cells were treated with Sunitinib for 48 h for indicated concentration. Increased sub-G1 fraction the Sunitinib -treated cells provided an estimate of apoptotic cells. **B**, Apoptosis detected by Annexin V-FITC/PI binding assay. Cells treated with Sunitinib for 48 h were stained with Annexin V and PI before subjected to FACS for analysis.

To investigate whether apoptosis is involved in the growth inhibitory effect of sunitinib malate, we determined its effect firstly by flow cytometry following PI staining and Annexin V-FITC/PI staining assay. As shown in Figure 2A, sunitinib malate treatment resulted in an accumulation of cells in the sub-G1 phase, especially with the T24 and BIU87 cell lines. After that, the most sensitive cell line, T24, was chosen to perform the Annexin V-FITC/PI staining assay for quantitative determination of apoptotic cells. Annexin V is usually used to detect the translocation of phosphatidylserine during early stages of apoptosis due to its high affinity for phosphatidylserine. Early apoptotic cells are positive for Annexin V-FITC only while late apoptotic cells are positive for both dyes. Viable cells should be stained negatively. As shown in Figure 2B, sunitinib malate treatment induced a markedly increase in the percentage of apoptotic cells in a dose-depended manner.

**Figure 3:**
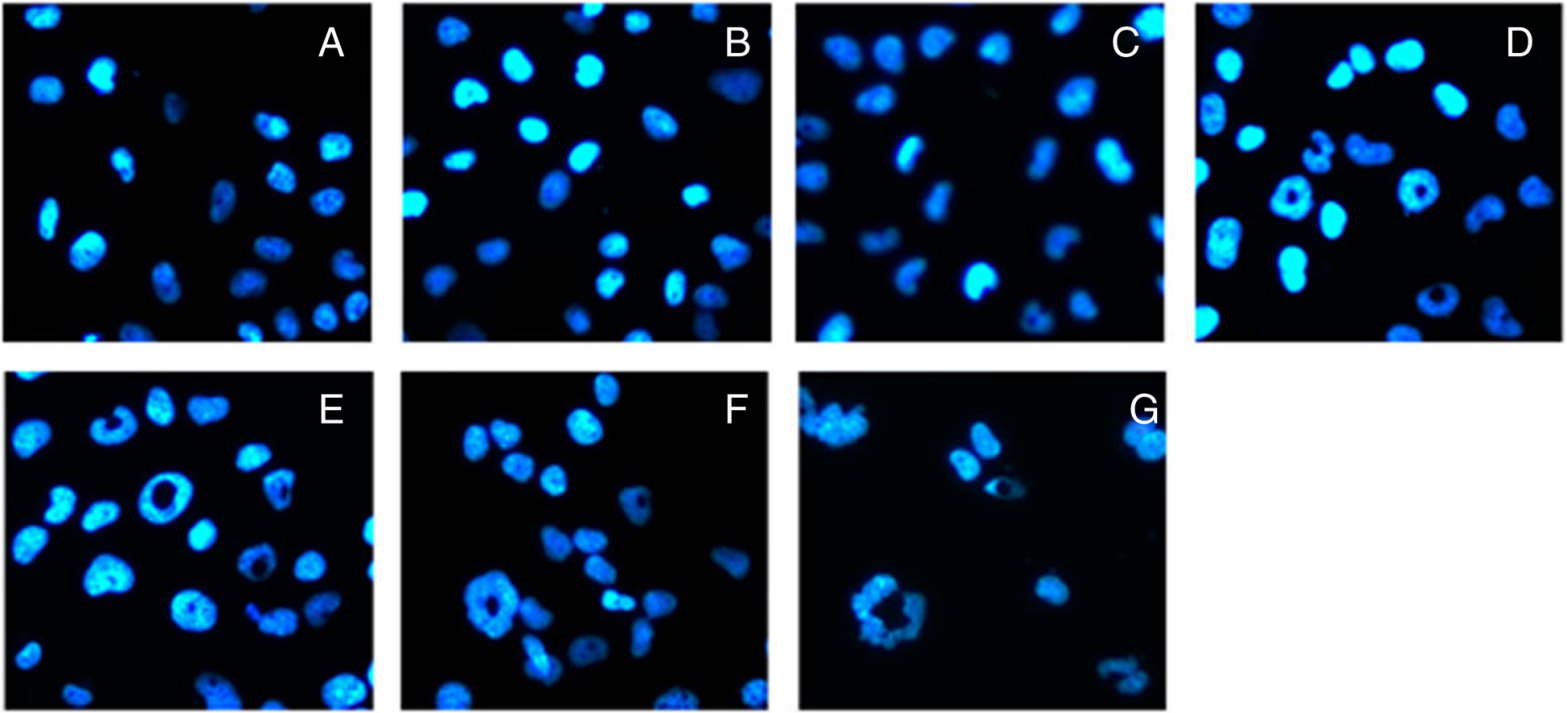
**Morphologic changes in T24 cells treated with sunitinib (A: 0 μmol/L; B: 0.3125 μmol/L; C: 0.625 μmol/L; D: 1.25 μmol/L; E: 2.5 μmol/L; F: 5 μmol/L; G: 10 μmol/L).** After exposure to different concentrations of sunitinib malate for 24 hours, the cells were stained with DAPI and examined using florescence microscopy (magnification 200 ×).

To further investigate its apoptotic effect, morphologic analysis via DAPI staining and fluorescence microscopy was employed for the T24 cell line. After 48 hours of exposure to sunitinib malate (0.3125-10 μmol/L), T24 cells showed unusual changes in their nuclei. As shown in Figure 3, at concentrations of 1.25 μmol/L or higher, small holes appeared in the nuclei of T24 cells and the holes were bigger with higher concentrations. In comparison, the nuclei of untreated T24 cells and those exposed to lower concentrations of sunitinib malate (0.3125 and 0.625 μmol/L) exhibited normal morphology.

**Figure 4:**
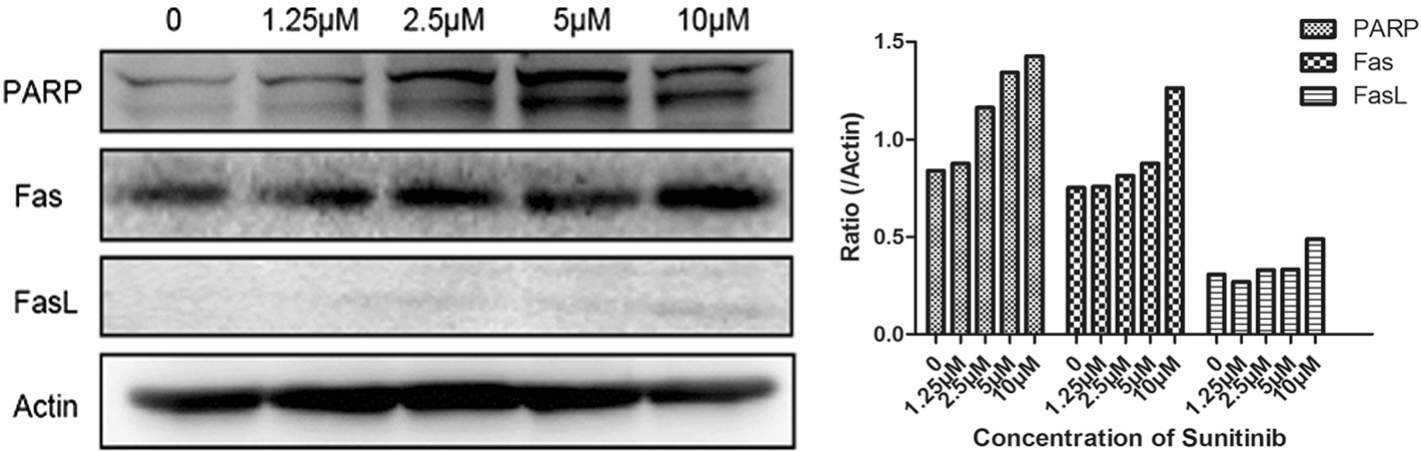
**Western blot analysis of protein expression of T24 cells treated with sunitinib malate.** T24 cells were exposed to sunitinib (1.25 μmol/L, 2.5 μmol/L, 5 μmol/L and 10 μmol/L) for 48 hours, and western blot analysis with antibodies specific for Fas, FasL, PARP and β-actin is shown. β-Actin levels are shown as an internal control.

It is well known that cellular apoptosis is associated with alterations in two main signaling pathways termed the extrinsic and intrinsic pathways. Yoon et al. [6] have shown that the intrinsic pathway is involved in the cell growth inhibitory effect of sunitinib malate, and protein expression in the extrinsic pathway, which is also known as the death receptor pathway, was also detected. Death receptors are members of the tumor necrosis factor (TNF) receptor gene superfamily that consists of more than 20 proteins, the best known of which is Fas (CD95), which has FasL (CD95L) as its corresponding ligand. As shown in Figure 4, the expression of FasL in T24 cells treated with sunitinib malate (1.25-10 μmol/L for 48 h) exhibited a significant concentration-dependent increase, but there was practically no change in the expression of Fas.

Cleavage of PARP is also an established and reliable apoptosis indicator downstream of caspase activation. As shown in Figure 4, T24 cells exhibited a concentration-dependent increase of PARP cleavage when exposed to sunitinib malate.

### Suppression of wound healing by sunitinib malate in T24 cells

**Figure 5:**
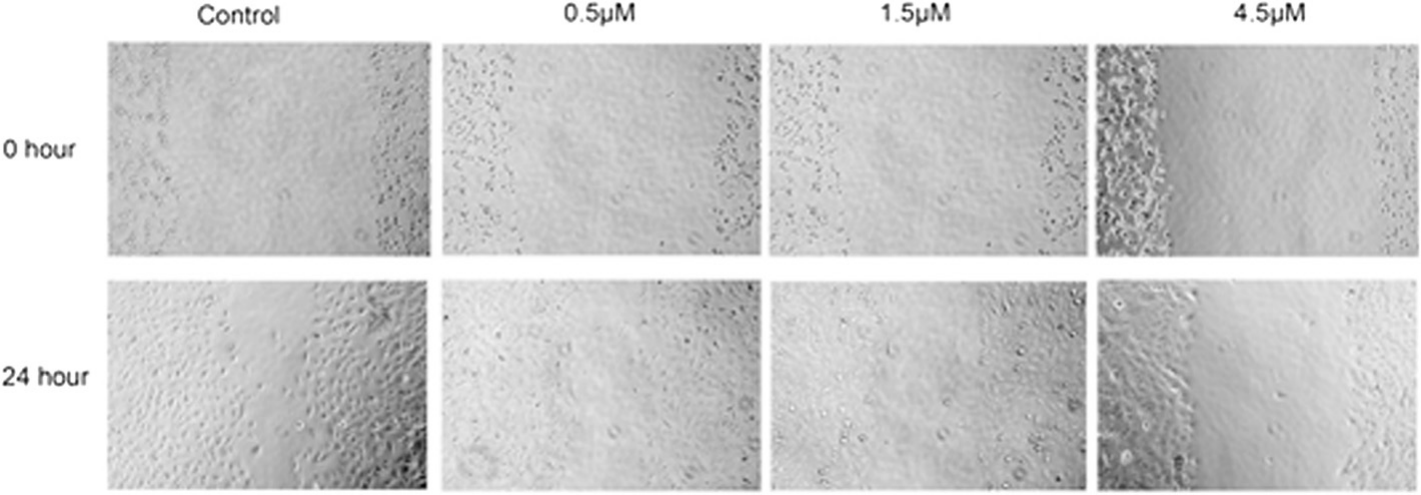
**Effect of Sunitinib on wound healing.** Confluent monolayers of T24 cells were mechanically wounded with the tip of a pipette. After 24 hours incubation with sunitinib with different concentration, images were taken under a phase-contrast microscope.

As it is well established that successful wound healing involves a number of processes, including cell migration and polarity, we performed a wound healing analysis to examine whether sunitinib malate affects these processes. As shown in Figure 5, sunitinib malate suppressed the wound healing process significantly in a concentration-dependent manner.

## Discussion

Sunitinib malate is a novel anti-angiogenic agent that has recently been demonstrated to improve progression-free survival in patients with metastatic renal cell carcinoma [7]. It is a small-molecule multi-RTK inhibitor that directly inhibits VEGFR, PDGFR, KIT, and FLT3. In patients with bladder cancer, the expression level of VEGF mRNA and the serum level of VEGF have been found to be associated with the cancer stage, grade, vascular invasiveness, and metastases [8-10]. It has also been shown that VEGF is abnormally over-expressed in TCC and promotes cancer malignancy [11]. These findings suggest a possible antitumor and anti-angiogenic effect of sunitinib malate in advanced bladder TCC. Thus far, however, there have been few studies of the antitumor effect and mechanism of action of sunitinib malate in bladder cancer [12].

In the present study, we firstly used MTT assay to explore the antitumor effect of sunitinib malate on 3 human bladder cancer cell lines. Although two of them, T24 and BIU87, originated from transitional urinary bladder carcinoma, and 5637 originated from GradeIIurinary bladder carcinoma, both of them showed high sensitivity to sunitinib malate therapy. In addition, we also compared sunitinib malate with another small-molecule multi-RTK inhibitor sorafenib and a conventional chemotherapeutic agent cisplatin. We found that both sunitinib malate and cisplatin exhibited a significant inhibitory effect against these cancer cells, but the cells were less sensitive to sorafenib. This finding points to the possibility of clinical application of sunitinib for advanced bladder cancer therapy.

Although sunitinib malate is a widely used drug with multiple targets, we did not examine its effect on targets such as VEGFR, PDGFR and KIT etc., in the TCC cell lines utilized in our study. What we were interested in is whether sunitinib malate can induce apoptosis and other characteristic changes in the cells. Flow cytometry analysis showed that sunitinib malate treatment resulted in accumulation of cells in the sub-G1 phase, especially with T24 and BIU87 cell lines, which could partially explain its growth inhibitory effect on these cells. Moreover, the morphologic analysis following DAPI staining showed an unusual change in the nuclei of T24 cells, a single hole, after exposure to sunitinib malate, which was probably an early apoptosis phenomenon.

Yoon et al. [6] have previously shown that sunitinib malate treatment significantly increased expression of the pro-apoptotic proteins Bax and Bad, indicating that the intrinsic apoptosis pathway is involved in the cell growth inhibitory effect of the drug. The concentration-dependent augmentation of FasL by sunitinib malate noted in our study suggests a possible involvement of the extrinsic apoptosis pathway in the antitumor action of the drug against TCC.

As discussed above, the cleavage of PARP is an established and reliable apoptosis indicator downstream of caspase activation, and is a fairly early event in apoptosis. PARP is a nuclear DNA-binding protein that detects DNA strand breaks and functions in base excision repair. However, once PARP is cleaved, it no longer supports the enzymatic DNA repair function, and there is increasing evidence that cleaved PARP may inhibit access to DNA by other repair enzymes. Several studies have indicated that PARP cleavage is detectable earlier than other events associated with apoptosis such as DNA fragmentation. Our study found that T24 cells exhibited a concentration-dependent increase in PARP cleavage when exposed to sunitinib malate which might partially explain the morphologic findings following DAPI staining.

To gain further insight into the effects of sunitinib malate on other characteristics of TCC, we examined its effect on cell migration and polarity via a wound healing assay and found that it suppressed the wound healing process significantly in a concentration-dependent manner. This finding further suggests its possible usage in the treatment of TCC.

In conclusion, we found that sunitinib malate exerted marked inhibitory activity against bladder cancer cells, and the cell growth inhibitory effect was related to induction of apoptosis. We also showed that sunitinib malate can suppress the motility of T24 bladder cancer cells. These findings point to the possible clinical application of sunitinib-based therapy for advanced bladder cancer.

## Abbreviations

DAPI: 4′,6-diamidino-2-phenylindole
DMSO: Dimethyl sulfoxide
FCM: Flow cytometry
FACS: Fluorescence-activated cell sorting analysis
FLT3: FMS-like tyrosine kinase-3 receptor
GC: Gemcitabine, cisplatin
GIST: Gastrointestinal stromal tumor
IC_50_: 50% inhibitory concentration
MTT: 3-(4,5-dimethylthiazol-2-yl)-2,5-diphenyltetrazolium bromide
MVAC: Methotrexate, vinblastine, doxorubicin, cisplatin
PARP: Poly adenosine diphosphate ribose polymerase
PBS: Phosphate-buffered saline
PDGFR: platelet-derived growth factor receptor
PFA: Paraformaldehyde
PI: Propidium iodide
RTK: Receptor tyrosine kinase
SDS-PAGE: Sodium dodecyl sulfate polyacrylamide gel electrophoresis
TCC: Transitional cell carcinoma
VEGFR: Vascular endothelial growth factor receptor.
